# Twenty years of childhood blindness: what have we learnt?

**Published:** 2008-09

**Authors:** Clare Gilbert, Mohammed Muhit

**Affiliations:** Professor, International Centre for Eye Health; Medical Advisor, Sightsavers International, UK.; Clinical Research Fellow, International Centre for Eye Health, UK.

**Figure:**
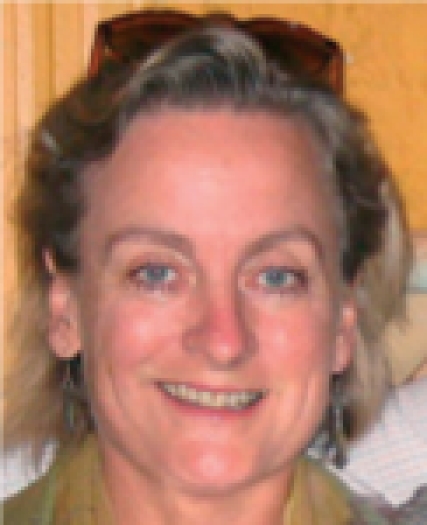


**Figure:**
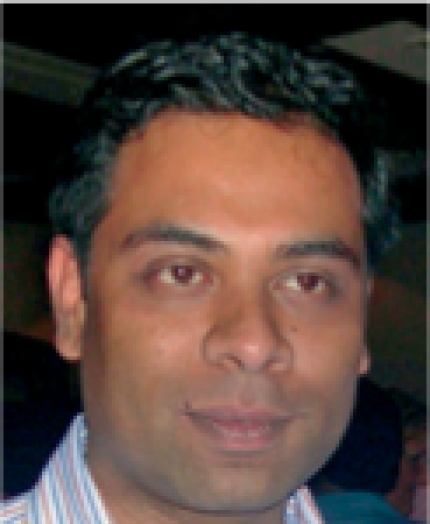


Over the last 20 years, much has been achieved in controlling blindness in children. Prior to the launch of VISION 2020, a number of international initiatives and programmes had raised the profile and increased interventions for child health and survival, which also had a positive impact on eye diseases and blindness in children, e.g. the Expanded Programme for Immunisation (EPI)(1974) and the Global School Health Initiative (1995). Since 2000, the United Nations' Millennium Development Goals have emphasised the need to promote child health and survival.

Since VISION 2020 was launched in 1999, controlling blindness in children has been a high priority. Child Eye Care Centres are being established with well-trained, well-equipped teams, particularly in Asia. Programmes for detecting babies with retinopathy of prematurity (ROP) are expanding in Latin America, India, China, and other countries in Asia. Many school-going children are having their visual acuity measured and those with refractive error are being provided with spectacles. Finally, there is improved availability of affordable consumables and equipment, such as paediatric low vision devices, small diameter intraocular lenses, and spectacles for children.

This article presents an overview of what we have learnt over the past twenty years and outlines some of the challenges we still have to face in order to control avoidable blindness in children and adequately support those with incurable visual loss.

## 1 Magnitude and causes

In 1988, there was little information on the magnitude and causes of blindness and visual impairment in children. At that time, most of the information on causes had come from examining children in schools for the blind and prevalence data were very limited. However, we have since made substantial progress, both in collecting data and in finding ways to estimate the magnitude and causes of childhood blindness.

In 1990, the World Health Organization (WHO) organised the first meeting of experts on the prevention of blindness in children; they estimated that there were 1.5 million blind children in the world.^1^ In 1997 WHO held a second meeting, during which the estimate was revised to 1.4 million: a new method was used to estimate the magnitude for countries where these data were not available, which was based on the association between under-five mortality rates and the prevalence of blindness in children. This method is still being used, as data from more recent population-based surveys confirm that the prevalence of blindness in children and under-five mortality rates correlate reasonably well. Under-five mortality rates are also being used as a proxy indicator of vitamin A deficiency in children.^2^

In 1990, experts agreed that corneal scarring, mainly from vitamin A deficiency and measles, was the major cause of childhood blindness in most low-income countries.^1^ However, as a simple classification of causes did not exist, the International Centre for Eye Health (ICEH) worked with WHO to develop a new system for classifying the causes of blindness in children, which was published in 1993.^3^ Data collected using this system, which has clear definitions, a standard recording form, and a data analysis package, is being used to compile a global database of causes.^4^ In 1997, it was estimated that 45% of blind children were blind from avoidable causes and that the pattern of causes varied widely between and even within countries.^4^ The following conditions were prioritised for control: corneal scarring, cataract, retinopathy of prematurity, refractive error (mostly myopia), and low vision.^5^ Other studies have shown that most blind children are either born blind or lose their sight before their sixth birthday. Novel methods have also been developed which can provide important information on the prevalence and causes of blindness in children, such as the use of local volunteers who act as key informants.^6^

## 2 Vitamin A deficiency and measles

During the 1980s, it was realised that vitamin A deficiency was an important cause of child mortality and that high-dose vitamin A supplementation significantly reduced child deaths, even in communities with low levels of clinical xerophthalmia. Intermittent high-dose vitamin A supplementation is an important public health intervention and approximately 500 million doses are given annually throughout the world at a cost of approximately US $1 per dose. As a result, the prevalence of vitamin A deficiency has declined in many regions of the world. There is also evidence that blindness in children due to corneal scarring has also declined. For example, in Uganda, 53% of all blind children born between 1951 and 1965 were blind from corneal scarring, compared with 14% for children born between 1980 and 1995.^7^ However, some communities are still affected by vitamin A deficiency today, such as those living in urban slums and poor communities in rural areas.

**Figure:**
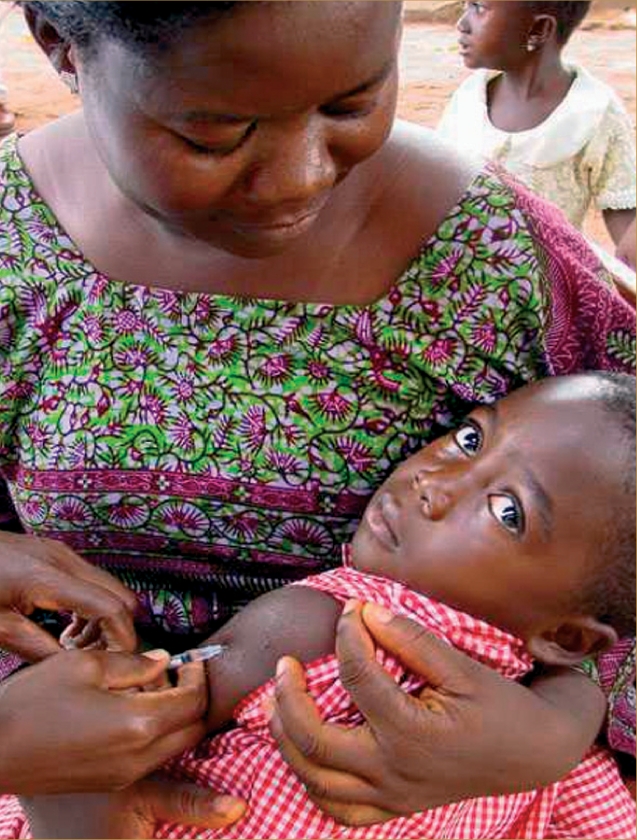
As a result of intense efforts to reduce under-five mortality rates over the last 20 years, the prevalence of corneal blindness in children has declined

Measles immunisation is another large-scale public health intervention to reduce child morbidity and mortality. Since the launch of EPI in 1974, coverage with measles immunisation has increased to target levels in most regions. The numbers of measles cases and measles-related deaths have declined as a consequence. Measles epidemics are now relatively rare and measles-related corneal blindness has also declined. As with vitamin A deficiency, there are still communities of children at risk, particularly in sub-saharan Africa, where the majority of measles cases and measles-related deaths now occur.

There is no reason to be complacent, as there is evidence that under-five mortality rates are no longer declining at the same rate as in earlier decades in the poorest parts of the world. Our role as eye care professionals is to advocate for children – when we see a child with corneal ulceration from vitamin A deficiency or following measles, we should not only treat the child, but we should also inform the relevant authorities so that they can improve their programmes.

## 3 Cataract in children

Because corneal blindness is declining in many countries in Africa and Asia, cataract is becoming a relatively more important cause of avoidable blindness. The management of cataract in young children has changed dramatically over the last 20 years. When intraocular lenses (IOLs) were first used in the late 1970s, they were thought not to be suitable for children. Over the last decade, smaller, high-power IOLs, suitable for children, have become available; surgical techniques and equipment have also evolved. Many paediatric ophthalmologists now insert IOLs in children as young as 12 months of age. However, this technique requires considerable expertise and a vitrectomy machine, as the posterior capsule and anterior vitreous have to be removed in young children. Long-term follow-up is also crucial to manage visual axis opacities and to ensure the child is given optimal optical correction and low vision devices, if required.

## 4 Retinopathy of prematurity (ROP)

In the early 1990s, it became apparent that an epidemic of blindness due to ROP was occurring in middle-income countries.^8^ There are three main reasons for this epidemic: neonatal care services have expanded and premature babies are surviving; levels of neonatal care are not always adequate to prevent ROP, particularly in larger, more mature babies; finally, not all babies at risk are being examined by an ophthalmologist and treated, if required. Recent evidence suggests that ROP is also emerging as a new cause of blindness in urban centres in India, China, and other countries in Asia. In these low- and middle- income regions, premature babies are also at risk of severe ROP with higher birth weights than is currently the case in industrialised countries. The implications of these findings are that neonatal care needs to be improved to prevent ROP and that examination of babies in neonatal units needs to include larger premature babies.

## 5 Refractive error in children

Little was known about the magnitude of vision loss due to refractive error in children until the late 1990s. Since 2000, WHO has worked with the National Eye Institute, USA, to undertake standard, population-based surveys of the prevalence of refractive error in all regions of the world among children aged five or seven up to 15 years. The findings show that refractive errors are more prevalent in children in Asian countries than in other regions, and that myopia, which increases with increasing age, is the commonest refractive error in older children. Myopia is also more common in children from urban areas than in those from rural areas. The current thinking is that myopia is caused by the influence of genes as well as environmental factors, and that outdoor activity may protect children from myopia. We also know from studies in Tanzania^9^ and Mexico, that a high proportion of children with refractive error identified in school eye health programmes do not wear the spectacles provided. All these studies provide important information for those planning school screening for refractive error.

**Figure:**
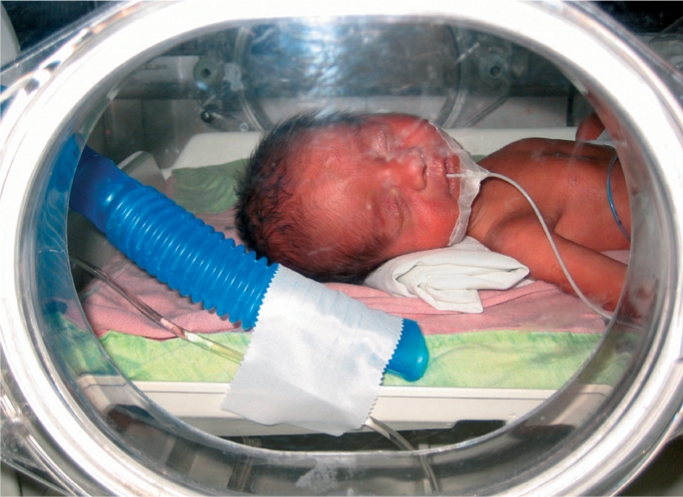
Premature baby in a neonatal intensive care unit. VENEZUELA

## 6 Functional low vision in children

Recent data from the studies of refractive error mentioned above suggest that the prevalence of functional low vision (corrected visual acuity in the better eye ranging from <6/18 to, and including, light perception from untreatable causes) is approximately twice the prevalence of blindness: there are almost 3 million children worldwide who have the potential to benefit from low vision care.^10^ It is, therefore, essential that low vision services be part of eye care services for children at all levels of service delivery.

## 7 Conclusion

The control of blindness in children is a VISION 2020 priority because the number of ‘blind person years’ resulting from blindness starting in childhood is second only to cataract. If VISION 2020 targets for children can be met, the global prevalence of blindness will have fallen from 7.5/10,000 children (in 1997) to 4/10,000 children by the year 2020. The number of children who are blind will decline to approximately 800,000.

Visual loss from refractive error and cataract is treatable, and blindness from vitamin A deficiency, measles, or ROP is potentially preventable. As eye care workers, we must provide the relevant information, services, and support to reduce blindness and visual impairment in children from these avoidable causes. We also need to ensure that children with incurable visual loss are not denied their equal right to education and to lives that are fulfilled and rewarding.

**Challenges for the future**

1. **Integrating eye health into broader child health initiatives**
  - International initiatives for the promotion of child health
  - Primary health care (e.g. kindergartens, Maternal Child Health Clinics, or microcredit schemes for women) for most of the preventable eye conditions.
2. **Tailoring services specifically for children**
  - Not treating children like small adults
  - Adopting a holistic, child-centred approach, which includes parents and carers
  - Developing and emphasising the sub-specialty of paediatric ophthalmology, from residency programmes through to service delivery
  - Planning services for a catchment population of 10 million people, rather than the 1 million used for adult blindness.
3. **Increasing uptake of services**
  - We have to be much more proactive in finding children who need treatment, particularly girls (e.g. by using local key informants)
  - We must try to make services affordable for children (free or greatly subsidised) and inform the community of how much is being charged
  - Health education for mothers is crucial: they should know how to prevent potentially blinding conditions and where they should go if their child has a problem
  - Health education for children, girls in particular, is equally important: they are the parents of the future
  - The current focus of school programmes on refractive error alone means that opportunities for health promotion and education are being missed. School-going children have siblings and parents at home.
4. **Recognising the importance of education and rehabilitation**
  - Many children attending eye care services have visual loss from untreatable conditions. It is our responsibility to ensure they are seen in low vision clinics and referred for education and rehabilitation
  - As avoidable causes are declining, a greater emphasis needs to be placed on preventing disability through education and rehabilitation.
5. **Ongoing research activities to improve programmes**
